# Photo-elasticity of silk fibroin harnessing whispering gallery modes

**DOI:** 10.1038/s41598-023-36400-0

**Published:** 2023-06-16

**Authors:** Nikolaos Korakas, Davide Vurro, Odysseas Tsilipakos, Thomas Vasileiadis, Bartlomiej Graczykowski, Annamaria Cucinotta, Stefano Selleri, George Fytas, Salvatore Iannotta, Stavros Pissadakis

**Affiliations:** 1 Foundation for Research and Technology-Hellas (FORTH), Institute of Electronic Structure and Laser (IESL), 70013 Heraklion, Greece; 2 Department of Materials Science and Technology, University of Crete, 70013 Heraklion, Greece; 3 Camlin Italy Srl, Strada Budellungo 2, 43123, Parma, Italy; 4 Theoretical and Physical Chemistry Institute, National Hellenic Research Foundation, 11635 Athens, Greece; 5 Faculty of Physics, Adam Mickiewicz University, ul. Uniwersytetu Poznańskiego 2, 61-614 Poznań, Poland; 6 University of Parma, 43121 Parma, Italy; 7 Max Planck Institute for Polymer Research, Ackermannweg 10, 55128 Mainz, Germany; 8 Institute of Materials for Electronics and Magnetism (IMEM), CNR, 43124 Parma, Italy

**Keywords:** Fibre optics and optical communications, Biomaterials, Microresonators, Imaging and sensing

## Abstract

Silk fibroin is an important biomaterial for photonic devices in wearable systems. The functionality of such devices is inherently influenced by the stimulation from elastic deformations, which are mutually coupled through photo-elasticity. Here, we investigate the photo-elasticity of silk fibroin employing optical whispering gallery mode resonation of light at the wavelength of 1550 nm. The fabricated amorphous (Silk I) and thermally-annealed semi-crystalline structure (Silk II) silk fibroin thin film cavities display typical Q-factors of about 1.6 × 10^4^. Photo-elastic experiments are performed tracing the TE and TM shifts of the whispering gallery mode resonances upon application of an axial strain. The strain optical coefficient K’ for Silk I fibroin is found to be 0.059 ± 0.004, with the corresponding value for Silk II being 0.129 ± 0.004. Remarkably, the elastic Young’s modulus, measured by Brillouin light spectroscopy, is only about 4% higher in the Silk II phase. However, differences between the two structures are pronounced regarding the photo-elastic properties due to the onset of β-sheets that dominates the Silk II structure.

Optical biomaterials offer numerous physical and chemical functionalities associated with their mechanical, segmentation, and surface chemistry properties. The former functions can accelerate the implementation of such materials into innovative and functional sensing, imaging, and actuating photonic devices^1–4^. An important feature of several protein-based biomaterials is the metastable-stable structure transition modifying the material’s optical^5,6^ characteristics. Bombyx Mori extracted silk is a natural biomaterial renowned for its applications in clothing and surgery. At the same time, its fibroin protein, in the regenerated form, has been widely studied for its use in optical circuits, drug delivery, and sensing components^7^. Due to its biocompatibility, mechanical strength, high optical transmission, and tailored wettability, silk fibroin is considered a backbone for developing wearable and implantable sensing devices^8,9^, allowing the realization of skin attachable components with readily accessible optoelectronic functions^10^. A significant issue arising for the realization of silk-based, skin-attached^11^, wearable photonic devices^12^ is that of photo-elasticity, namely, how much the refractivity and birefringence of the silk fibroin will change under mechanical stimulation. For example, the operation characteristics of optical devices in adhesive contact with human skin tissue can depend on the mechanical forces directly exerted at the interface region. On the photo-elasticity of silk, the limited literature primarily refers to the strain-induced birefringence in pristine silkworm filaments^13,14^, whereas, to the best of our knowledge, there is no report on regenerated silk fibroin. Photo-elasticity correlates mechanical and optical properties of a material reflecting local structure onto macroscopic response. The specific local structure of regenerated silk fibroin, where protein structures such as random coils, β-sheets, and α-helixes conglomerate, render a photo-elasticity study particularly important.

Herein, we report on silk fibroin's photoelastic properties, employing whispering gallery mode (WGM) light resonation in cylindrical resonators cast onto glass fiber supporting beams. Owing to the high Q-factor, the polarization-sensitive, modal dispersion of light confinement facilitated through WGM resonation^15^ allows the detection of minimal birefringence changes yielding the stress-optical coefficient and enabling a correlation to the molecular polarizability of soft materials^16^. Micrometric-size silk fibroin cylindrical cavities are first formed in Silk I (metastable) and then transformed to Silk II (stable), allowing an in-situ investigation of the effect of the structure on the photoelastic behavior of silk fibroin. The formation of β-sheets in Silk II simultaneously modifies the optical and mechanical properties of the silk fibroin, dominating its photoelasticity. While silk fibroin has been used before in the fabrication of cylindrical WGM cavities onto planar substrates^17^, the application of WGM resonation in the study of the photo-elasticity of a protein-based biomaterial is a new approach, implemented explicitly for the case of silk fibroin. For a detailed insight into the origin of the photoelastic differences between the amorphous and semi-crystalline structures of silk fibroin, the elastic Young’s moduli and Poisson’s ratio of both phases are measured at zero strain by the non-contact, non-invasive Brillouin light spectroscopy (BLS). BLS enables the de-coupling of the mechanical properties of the two silk structures from their optical refractivity/polarizability properties^18^. In view of the unexpectedly very similar Young’s moduli, the photo-elasticity of silk fibroin is primarily driven by the optical properties of the β-sheet conformations formed in excess in the semi-crystalline Silk II, with the Silk I being less birefringent due to low β-sheet content and water plasticization.

## Experimental

**Figure 1:**
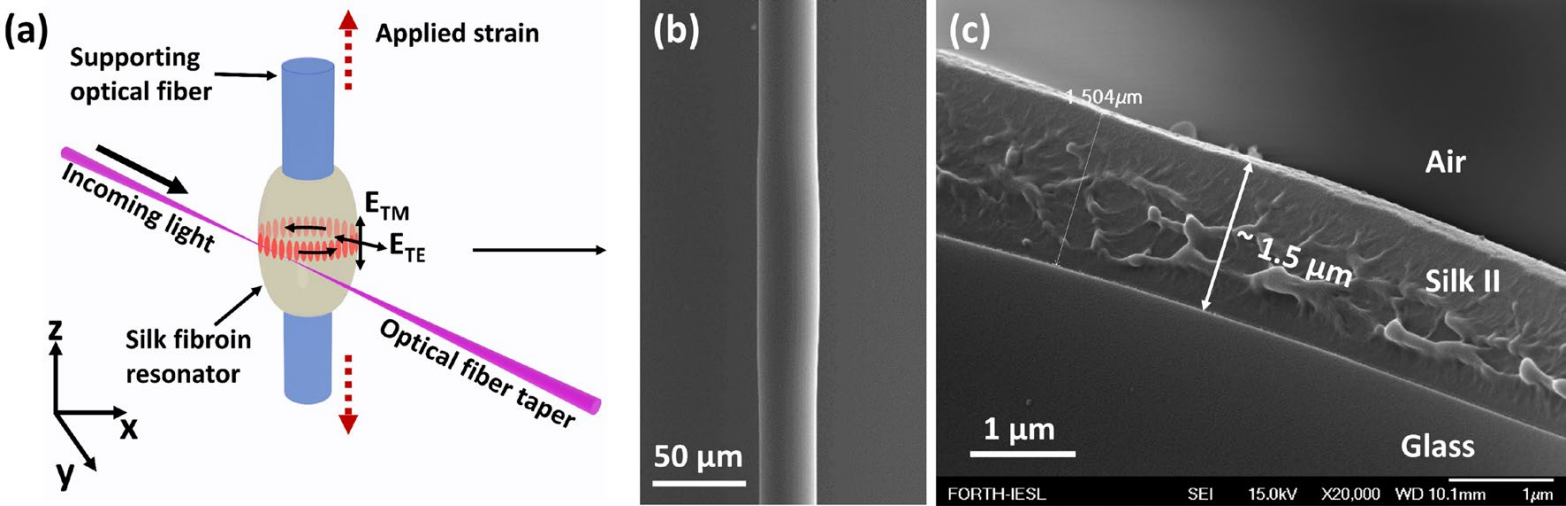
(**a**) Sketch of the experimental setup for spectrally characterizing the silk fibroin resonating cavities. (**b**) SEM picture of a typical silk fibroin cavity formed onto a silica optical fiber taper, after being annealed to obtain the Silk II structure. (**c**) Transversal SEM picture of a radial cross-section of a ~ 1.5 μm thick Silk II fibroin film overlaid onto an underlying ~ 87 -μm- diameter silica glass optical fiber taper.

The silk fibroin optical micro-resonators were formed onto ~ 30 μm diameter silica glass optical fiber tapers. The intrinsically hydrophobic silica glass SMF-28 optical fiber tapers turned hydrophilic, employing a preparatory 193 nm excimer laser irradiation process^19^ and achieving an even shape cavity formation from a silk-water solution. The silk fibroin used was extracted from row Bombyx Mori cocoons following the original Rochwood’s protocol; the extracted fibroin was dissolved into an 8% (wt/vol) water solution (See “Methods”). Almost cylindrical shape cavities of a typical height of ~ 100 μm and diameters of ~ 33 μm were fabricated onto SMF-28 optical fiber tapers (Fig. 1a).

A silk fibroin over layer of ~ 1.5 μm thickness is evenly attached onto the glass surface (see Fig. 1b). The cross-sectional SEM picture of Fig. 1c, where the morphology of the film exhibits observable spatial variations^20^, is attributed to local β-sheet conglomeration. To suppress higher order WGM excitation and hence, obtain a few notch spectra^21^, a 2.2 μm diameter tapered optical fiber was chosen for exciting the WGMs inside the silk fibroin optical cavity, in close proximity mode. Amplified spontaneous emission (ASE) from an Erbium doped fiber amplifier (EDFA) was coupled into the excitation optical fiber taper, while the WGM spectral signature was traced through the same fiber (the other end), connected to a polarizing optical fiber, while using an optical spectrum analyzer (see “Methods”). For quantifying photo-elasticity changes in the silk fibroin material, the composite system of the silk fibroin-WGM cavity/silica beam is firmly glued onto a specialty design metallic form; then it is subjected to controllable strain along the z-axis (see scheme in Figs. 1a and S1 in the Supplementary part), while simultaneously monitoring the corresponding spectral shift of WGM notches. A DC servo motor actuator was used for the precise elongation of the optical fiber with the silk fibroin cylinder (see Supplementary part Fig. S1).

The Young’s modulus of Silk I and II fibroin films (5 μm and 13.5 μm thick) cast on sodalime glass substrates is computed from the sound velocity measured by Brillouin light scattering (BLS), using 532 nm laser radiation (see “Methods” and Supplementary part Sect. 2, Fig. S2). BLS can also be used to extract the relative values of the Pockel’s coefficients in Silk I and II cast on sodalime glass substrates, from the intensity of the Brillouin peaks (again see “Methods”). Finally, the crystallinity of Silk II structure is verified by Raman measurements carried out (see Fig. S6), focusing on the 1085 cm^−1^ peak associated with the β-sheet formation^22,23^.

## Theoretical formulation

For the silk fibroin WGM resonator, a purely cylindrical symmetry was assumed, which is a good approximation for modal excitations within its middle section. By staying far from side-located turning points^24^ we could examine only the radial *(l)* and azimuthal *(m)* modes for the TE or TM polarization states (Fig. 1a)^25^. The modal index *l* gives the number of electric field intensity maxima in the radial direction, and *m* is the azimuthal modal index, describing the number of wavelengths of the electrical field along the horizontal meridian of the cylindrical shape resonator. The strain-induced spectral shifts in the TE and TM WGMs notches are ascribed to alterations of both the resonator radius (Δr) and the refractive index (Δn_TE, TM_) through the relationship:

1 $$\frac{{\Delta \lambda_{TX} }}{{\lambda_{TX} }} = \frac{\Delta r}{r} + \left( {1 - C_{TX} } \right)\frac{{\Delta n_{TX} }}{{n_{TX} }}$$

where Δλ_TX_≡ λ_TX_(ε) − λ_TX_(0) and Δn_TX_≡ n_TX_(ε) − n_TX_(0) (index X stands for either E or M), and ε denotes the applied strain along the z-axis of the silk fibroin/silica beam system. C_TX_ is a correction factor compensating for inherently differential slopes holding for TE and TM polarizations of equal order WGM modes^15^, calculated from the analytical formulation for resonation in cylindrical cavities. For the specific parameters of our experiment, C_TE_ = 5.06 × 10^–2^ and C_TM_ = 2.64 × 10^–2^.

In the Silk I amorphous structure, we consider silk fibroin as an isotropic, modest birefringence optical material. We also treat Silk II (semi-crystalline) as an isotropic material since the crystallinity range of the dispersed β-sheets in Silk II fibroin (~ 2.6 × 3.2 × 11.6 nm) is manifold shorter^26^ than the propagating wavelength. This assumption allows us to use the contracted photoelastic tensor for isotropic materials, with inverse symmetry rotation simplifications applied in the Pockel’s coefficients^27^. Application of longitudinal strain through the supporting optical fiber beam to the silk fibroin cylindrical micro-resonator (Fig. 1a) generates uniaxial anisotropy, and the refractive index perturbation is different for the axial (Δn_z_), and transversal direction t analyzed along axes x and y (Δn_t_)^28^, as according to the relationships:

2 $$\frac{{\Delta n_{t} }}{{n_{0} }} = \frac{{\Delta n_{TE} }}{{n_{0} }} = - \frac{{n_{0}^{2} }}{2}\left[ {p_{12} \varepsilon_{z} - \left( {p_{11} + p_{12} } \right)\varepsilon_{t} } \right]$$

3 $$\frac{{\Delta n_{z} }}{{n_{0} }} = \frac{{\Delta n_{TM} }}{{n_{0} }} = - \frac{{n_{0}^{2} }}{2}\left[ {p_{11} \varepsilon_{z} - 2p_{12} \varepsilon_{t} } \right]$$

Here n_0_ denotes the isotropic refractive index (assumed almost identical along the t and z directions), ε_t, z_ are the strains, and p_ij_ are the Pockel’s elasto-optic coefficients. According to Eqs. (1), (2) and (3), the strain-induced birefringence $\frac{{\Delta n}_{strain}}{{n}_{0}}=\frac{{\Delta n}_{TM}}{{n}_{0}}-\frac{{\Delta n}_{TE}}{{n}_{0}}=\frac{{\Delta \lambda }_{TM}}{{\lambda }_{TM}}-\frac{{\Delta \lambda }_{TE}}{{\lambda }_{TE}}$ (deployed per polarization component TE and TM) leads to the optical strain coefficient ${K}^{^{\prime}}\equiv \frac{{\Delta n}_{strain}}{\left({\varepsilon }_{z}{+\varepsilon }_{t}\right)}=\frac{{n}^{3}}{2} ({p}_{12}-{p}_{11})$, with corresponding stress optical coefficient $K\equiv K{^{\prime}}\frac{1+v}{2E}$ where ν is the Poisson’s ratio, E is the Young modulus of the silk fibroin, and K’ is the strain-optical coefficient. Therefore, by knowing the spectral shift per polarization component of a fixed order WGM notch and the strain distribution on the silk fibroin cavity per axis, we can evaluate Pockel’s coefficients p_11_ and p_12_ and, correspondingly, the strain and stress optical coefficients of the material. The principal stresses along the three axes were computed with finite element method studies of mechanical strain using commercial software (COMSOL Multiphysics) (see Supplementary part, Sect. 5). The ε_z_ strain values used in those calculations were directly deduced from the experiment.

## Results

Several cylindrical cavities of silk fibroin, similar to the one shown in Fig. 1b, were cast from 8% (wt/vol) silk fibroin aqueous solution, using the process described in Sect.  2, and characterized with WGM resonation.

**Figure 2:**
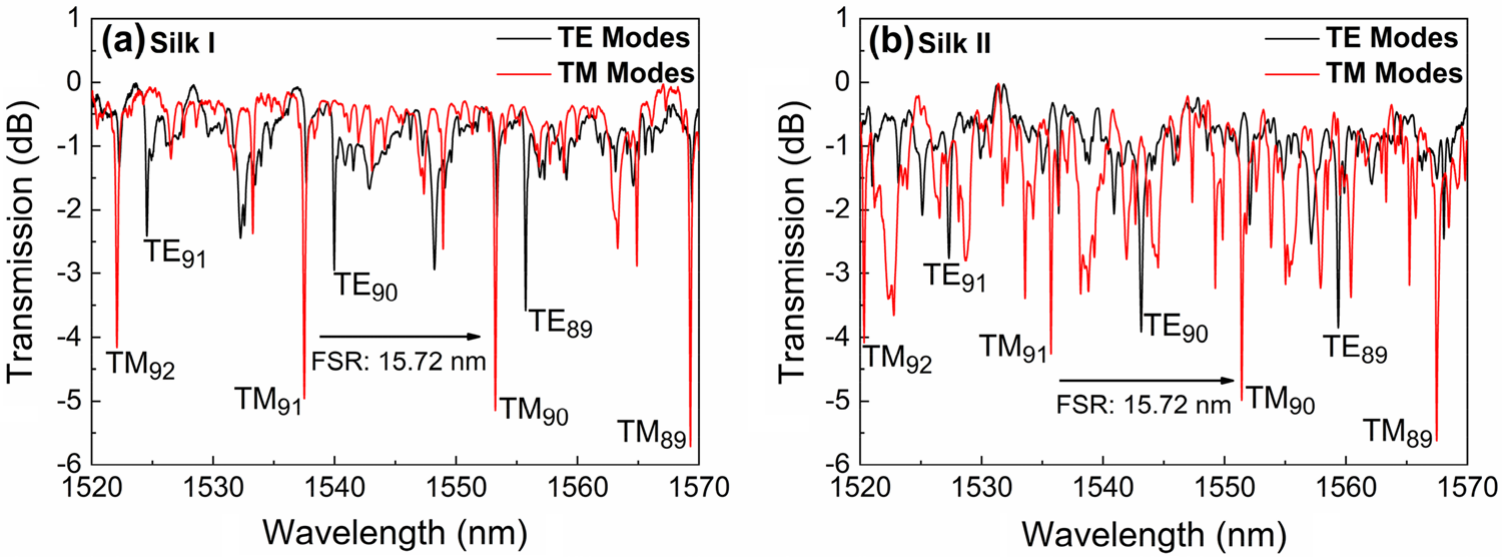
Transmission WGM spectra for a 32 μm diameter silk fibroin cavity, for TE (black) and TM (red) polarizations, as cast (Silk I) and after annealing at 180 °C (Silk II). The modal allocation presented above refers to modes with radial order l = 1.

Experimental spectral data of WGM resonances for both polarizations over a broad wavelength range for a silk fibroin resonator as cast (Silk I) are depicted in Fig. 2a and after thermal annealing process (Silk II) for the same cavity, are presented in Fig. 2 (b); these spectral data refer to the cavity depicted in Fig. 1b. The highest quality factors Q measured for the cavity of Fig. 1b for TM_89_ were 1.35 × 10^4^ for Silk I, and 1.61 × 10^4^ for Silk II, under a Lorentzian function fitting^29^. It is anticipated that the annealing process improves silk fibroin homogeneity and slightly increases its refractive index^30^. These Q-factor values rest below those reported by other groups on Silk I WGM cavities; however, they are measured in closed-shape cavities of better radial symmetry than those casted here^17^. The experimental free spectral range (FSR) (${\approx \lambda }^{2}/2\pi rn$) is measured at ~ 15.72 nm for both Silk I & II structure cavities for TM polarization and l = 1 radial modes; similar FSR figures hold for TE polarization for Silk I (15.44 nm) and Silk II (15.85 nm) state. Given that the difference in the TM modes refractive index between Silk I and Silk II structures is typically Δn ~ 0.005, a radius difference of Δr ~ 5 nm for the Silk II cavity is deduced as a consequence of the annealing process (about 0.34% shrinkage in the radial direction of the cavity^31^). The as-cast (residual) birefringence Δn_initial_ of the Silk I fibroin cavity was estimated from the wavelength positioning of the two polarization notches for a fixed azimuthal/radial order compared to simulated ones (see Supplementary part, Sect. 3) to be ~ 0.5 × 10^–3^. For the annealed Silk II fibroin cavity, Δn_initial_≡ Δn_ΤM_ − Δn_ΤE_ ~ −5 × 10^–3^. Thus, the refractive index difference Δn_initial_ was found to have an opposite sign and a tenfold larger absolute value in Silk II than Silk I. This difference in residual birefringence is attributed to the thermally induced β-sheet formation and their orientation inside the cavity volume of Silk II^32^.

The WGM resonation was described with full-wave eigenvalue simulations^33^ using COMSOL Multiphysics (see Supplementary part). Before employing the numerical simulations in the silk fibroin cavities under study, their accuracy was verified in an archetypical cylinder resonator geometry by comparing the simulation results against rigorous analytical solutions^34^ (see Supplementary part, Sect. 3). In the simulations, the silk fibroin cavity was considered perfectly cylindrical $(\partial /\partial \mathrm{z}=0$) and treated as a two-dimensional resonant structure. This is justified by the slowly-varying geometry of the cavity along the z axis, and the fact that modal excitations occur within its middle section, far from side-located turning points. The free-spectral range of first-radial-order resonances is found to be ~ 16 nm, in agreement with experimental data (see Fig. S3 for more details). Mode allocation for the first-radial-order modes under the cylindrical approximation is depicted in Fig. 2. The interstitial spectral features in Fig. 2 are associated with the excitation of higher-radial-order modes (see Fig. S3). The distribution of light in the two materials can be calculated based on the rigorous finite element eigenvalue simulations. More specifically, light confinement was assessed by integrating the stored energy density inside the different material regions as a post-processing step. This is an important calculation for determining the contribution of the elasto-optic parameters of the silica glass. For the TE mode of Silk I with azimuthal order m = 89, depicted in Fig. S3c, only 2.1% of the total stored energy resides in the silica glass, 97.1% in the silk layer, and the remaining 0.8% in the surrounding air. The corresponding percentage in silica for the first-radial order TM mode with m = 89 is quite similar to the above (1.2%). As a result, for the first-radial-order modes that are excited in the structure (see Fig. 2), almost all the injected light intensity will be waveguided through the silk fibroin region. Therefore, it can be fairly concluded that the contribution of the silica glass to the overall photoelastic behavior is negligible and can be ignored.

**Figure 3:**
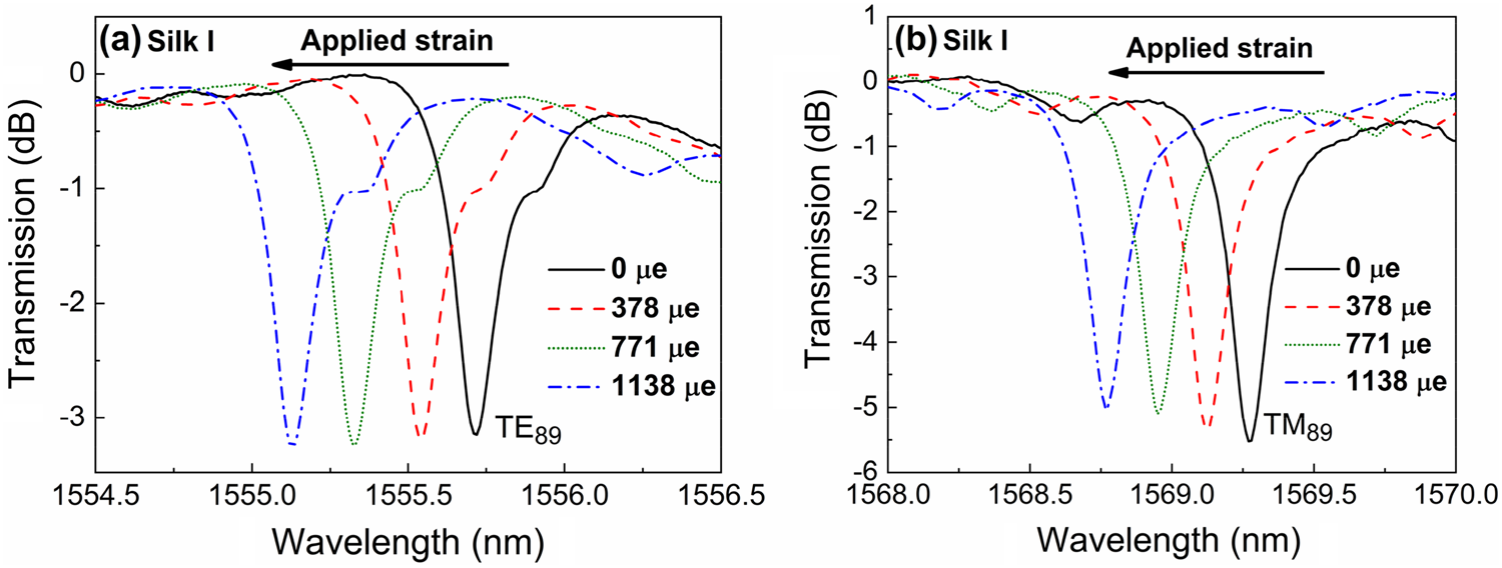
Transmission spectra of the TE and TM WGMs with modal order m = 89, l = 1, confined inside the silk fibroin cavity for Silk I state, under the applied strain (ε_z_) along the longitudinal axis of the supporting optical fiber.

From the above modal simulations, it was decided that all strain birefringence measurements should be focused on the spectral examination of first-order-radial modes. While higher radial order modes have also being experimentally traced (yet not presented here), their spectral behavior (low Q-factor and modal splitting versus strain application) rendered their interpretation laborious and of limited accuracy. The as-cast (Silk I) fibroin cavity was subjected to controllable longitudinal strain, and the spectral data of the TE and TM WGM of modal order m = 89, l = 1 are presented in Fig. 3.

**Figure 4:**
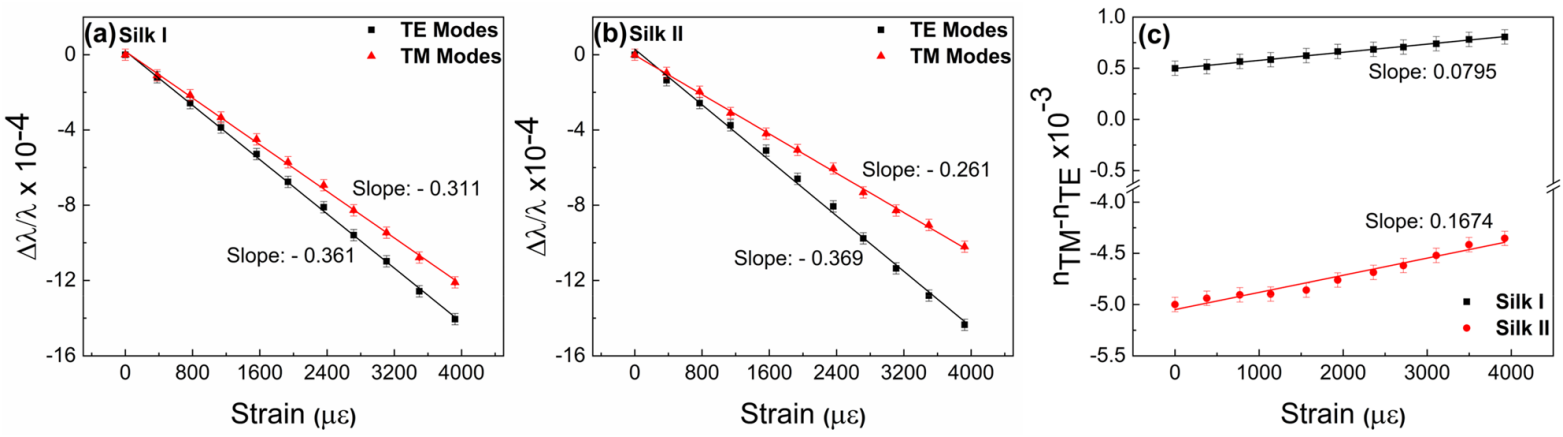
(**a**) and (**b**) Normalized WGM wavelength shift versus longitudinal strain (ε_z_) for TE and TM polarizations, as were experimentally measured. (**c**) Strain-induced birefringence. Both silk fibroin resonating cavities in the Silk I and II states are considered.

The WGM notches for both polarizations undergo a blue frequency shift as the applied strain increases in agreement with previous investigations in other cavity materials and geometries^15,35^; the WGM spectra for this cavity in the Silk II structure (presented in Supplementary, Fig. S4.) for the m = 89 modal order is of similar shape without significant differences from those presented in Fig. 3. The corresponding spectral shifts of the TE and TM WGMs of the silk fibroin cavity versus applied strain, as-cast (Silk I) and after thermal treatment (Silk II), are presented in Fig. 4, with the subsequent strain-induced optical birefringence emerging, being presented in Fig. 4c. For the strain conditions applied in the fibroin cavity for the Silk I and II structure, the dependence of the WGM spectral shifts versus elongation appeared linear and reversible; thus, no soft material detachment took place at the glass interface. Also, the slopes $\frac{\mathrm{\Delta \lambda }}{\mathrm{\lambda \varepsilon }}$ for the TE and TM modes, rest within the same order of magnitude as those obtained for other soft materials with similar Young’s modulus^16^. The data of Δλ/λ versus strain ε of the fibroin cavity appended in Fig. 4c reveal a quite interesting characteristic: the slopes $\frac{\mathrm{\Delta \lambda }}{\mathrm{\lambda \varepsilon }}$ of the WGM for TE polarization are almost identical for Silk I and II, while the slope of the TM polarization referring to Silk I is ~ 15% greater than that of Silk II. The latter is a consistent finding, observed in all five silk fibroin cavities examined in both Silk I and II, indicating the strain-induced birefringence in the specific silk fibroin cavities is directional and affects primarily the TM polarization component that is parallel to the longitudinal axis of the supporting optical fiber taper.

Figure 4c consolidates that the fibroin WGM cavity is more birefringent and strain-induced birefringent in the Silk II than the earlier Silk I structure; this purely optical behavior clearly reflects the fundamental protein re-arrangements induced by the thermal annealing process. The birefringence data of Fig. 4c are used for evaluating the Pockel’s coefficients p_11_ and p_12_ and that of the strain-optical coefficient K’ after estimating the strains exerted on the silk fibroin WGM cavity using a numerical 3D strain–stress analysis (see Fig. S5). The optical, mechanical, and photoelastic parameters of Silk I and Silk II fibroin films obtained from the WGM experiments are appended in Table 1. To compute the stress optical coefficient the K, it is necessary to know the Poisson’s ratios and Young’s moduli for the two different silk fibroin states (Eq. 2 & 3). These two physical parameters in Silk I and II states were also obtained from BLS experiments using films with similar thickness to those forming the WGM cavity (see “Methods” and Table 1). As in this work, previous investigations of Silk fibroin with BLS have also detected subtle differences in the longitudinal modulus between the amorphous and crystalline structures close to room temperature and above^36^. Importantly, Young’s moduli for the Silk I and II structures exhibit very similar values with the same Poisson’s ratio, 0.33 ± 0.01.

**Table 1:** Experimentally obtained elasto-optic properties of Silk I and II WGM cavities.

	Initial birefringence	Young modulus (GPa)	Pockel’s coefficients		Strain optical Coeff. K’	Stress-optical coeff.K (brewster)
			p_11_	p_12_		
Silk I	0.5 × 10^–3^ ± 7 × 10^–5^	9.34 ± 0.05	0.098 ± 3 × 10^–3^	0.132 ± 4 × 10^–3^	0.059 ± 9 × 10^–3^	8.1 ± (0.7)
Silk II	− 5 × 10^–3^ ± 7 × 10^–5^	9.69 ± 0.05	0.048 ± 3 × 10^–3^	0.120 ± 4 × 10^–3^	0.129 ± 9 × 10^–3^	17.0 ± 0.7

The polarized and depolarized BLS intensities obtained, respectively, from the longitudinal and transverse acoustic phonons in Silk I and II (see “Methods” and Supplemental material Sect. 2) yield the ratio values: ${p}_{12}^{silk I}/{p}_{12}^{silk II}=1.32$, ${p}_{11}^{silk I}/{p}_{11}^{silk II}=1.31$ and from ${p}_{44}=\left({p}_{11}-{p}_{12}\right)/2$, ${p}_{44}^{silk I}/{p}_{44}^{silk II}=1.23$. In the BLS experiment, the intensity of the longitudinal and transverse phonon peaks is controlled by the isotropic (∝ *p*_12_) and anisotropic (∝ *p*_44_) components of the segmental polarizability tensor, respectively^37^. Unexpectedly, the depolarized BLS intensities imply that the optical anisotropy per optical axis in the amorphous Silk I is about 20% higher than in its semi-crystalline form II. Notably, the WGM experiment gives ${p}_{12}^{silk I}/{p}_{12}^{silk II}$=1.1 (at 1550 nm) (Table 1) in satisfactory agreement with the isotropic BLS experiment (at 532 nm) considering possible refractive index dispersion effects. On the contrary, ${p}_{44}^{silk I}/{p}_{44}^{silk II}$= 0.47 from the WGM is about half the ratio value from the depolarized BLS experiment suggesting an anticipated higher optical anisotropy per optical axis in the semi-crystalline Silk II than in the amorphous Silk I. While both WGM and isotropic BLS experiments indicate very similar isotropic segmental polarizability in the fiber and film samples, their optical anisotropy per optical axis seems different. This is thoroughly discussed in the next section.

## Discussion

The amorphous Silk I and semi-crystalline Silk II have the same chemical stoichiometry but different refractive and photoelastic properties, as revealed from the WGM of thin film cavities. Silk II reveals a pronounced optical birefringence, with the higher refractive index component measured along the longitudinal axis of the WGM cavities. However, Silk II is characterized by Young’s modulus only slightly higher than Silk I. Hence the transformation of Silk fibroin from the amorphous to the semi-crystalline structure affects predominantly the optical birefringence, Δn_TM-TE_, (by an order of magnitude) and the stress optical coefficient, K (by more than 50%), but only marginally (by 4%) the elastic material response. These findings demonstrate that segmental orientation is a fundamental quantity for photo-elasticity. This notion is corroborated by the different ${p}_{44}^{silk I}/{p}_{44}^{silk II}$ in the fiber and film samples used in WGM and BLS experiments.

Silk I in a disordered structure includes some α-helix and random coil conformations and substantially less of β-sheet crystalline domains, moreover preserving a reasonable water content, which acts as a plasticizer^20^. The basically amorphous structure of Silk I is also confirmed by its low initial (residual) birefringence (Table 1), a feature that is mostly found in atactic polymers^38,39^. The photo-elasticity figures for Silk I are similar to those holding for bulk polymers; for comparison, the strain and stress-optical coefficients of polystyrene are ~ 0.01 and ~ 12Br, respectively, while assuming E = 3.4GPa, significantly lower than for the E value in Silk I, with similar Poisson’s ratio. The high E values of Silk I and II imply dense local packing, as suggested by the relatively high density ($1450 \mathrm{kg}/{\mathrm{m}}^{3}$)^40^.

For Silk II the polarization splitting and birefringence data are illustrative of the structural transformation of the Silk II structure. The annealing process in ambient air -which is the one followed in our experimental work-, gradually dehydrates the Silk I material, promoting the formation of β-sheets, for achieving Silk II form^32^. The slow temperature increase, breaks the intramolecular hydrogen bonds in Silk I structure and the molecules rearrange in the more stable, high crystalline β-sheets forms, without including endangering oxidation^41^. However, this structural crossover can impact the segmental orientation while has subtle effects on the elastic modulus suggesting similar local packing for Silk I and Silk II. Furthermore, the segmental orientation reflected in the value of the Pockel’s coefficient p_44_ should be local as suggested by the isotropic elasticity in the BLS experiment with the two moduli in- and cross-plane being the same. For a natural silk fiber system, specifically that of Nephila Pilipes spider silk fiber^42^, the elastic Young’s modulus is strongly anisotropic due to the chain stretching along the fiber axis. In fact the experimental E for both Silk film samples is very close to E value for the Nephila pilipes spider silk fiber normal to the fiber axis being about twice lower than along the fiber axis^42^.

The different TM WGM polarization Δλ/λ slopes of Fig. 4 for Silk I and II denote that the strain induced birefringence cannot be explained by the high stiffness of the β-sheets^26^, considering that the two fibroin structures have very similar mechanical properties. Instead, the clear disparity in the birefringence and stress optical coefficient is mainly due to ${p}_{11}$, which relates to the segmental optical anisotropy of the two Silk structures. Thus, the origin of large optical anisotropy in Silk II is attributed to the number and orientation of the β-sheets, and also the stretched chains linking these nanocrystals compared to Silk I. This feature is specifically captured with the WGM resonator experiments and remains elusive in the elastic Young’s modulus for the thicker film samples of the BLS experiment.

## Concluding remarks

We have investigated the photoelastic properties of Silk fibroin using whispering gallery mode resonation, offering high accuracy and significant insight in studying soft-materials. We propose that the photo-elasticity of Silk II is predominantly related to the high optical polarizability properties of the β-sheets and their strain induced alignment, rather than the mechanical properties of the annealed fibroin. Unexpectedly, the elastic Young’s moduli of Silk I and Silk II obtained by Brillouin light spectroscopy are very similar. The significance of β-sheets and their impact on the optical properties of Silk II have been illustrated in our study, paving the way for additional investigations. The use of external stimulations (i.e. high voltage poling) for tuning the formation and orientation of β-sheets emerges as a promising approach for developing optical biomaterials with high polarizability properties, for linear or non-linear^43^ optical operations. The β-sheet dominated refractivity and photo-elasticity of Silk II can be further fruitfully exploited in the development of planar or fiber guided wave structures, characterized by exotically high birefringence^38^, for use in polarization selective passive and active photonic devices; also, the elastic and inelastic light scattering properties of Silk II deserve further attention.

## Methods

### Silk fibroin extraction

Silk Fibroin (SF) was extracted from row Bombyx Mori cocoons following the original Rochwood’s protocol. Cocoons were sliced, then boiled in a 0.02 M sodium carbonate (Alfa Aesar) solution for 30 min in order to remove the glue-like cladding made of sericin. Degummed fibroin obtained upon separation from sericin was then washed three times in highly pure water for 20 min for removing eventual sericin excess; then, left to dry overnight under hood. Degummed fibroin then dissolved in 9.3 M lithium bromide (Alfa Aeser) solution for 4 h in a conventional oven at 60 °C, forming an amber-like solution. After cooling down, this solution was dialyzed for 48 h in a dialyzer cassette (Slide-A-Lazer 3.5 KDa, Thermo Fisher) against ultra-pure water to remove salts. After dialysis, the SF aqueous solution was centrifuged twice at 9000 rpm for 20 min at 4 °C to remove the unsolved portion of fibroin; a ~ 8%( wt/vol) solution has been accordingly produced and storage at 4 °C in the fridge prior to use.

### Silk fibroin cavity preparation

Optical fibers were thermally tapered down to ~ 30 μm diameters with the use of a GPX-3000 Vytran glass fiber processor. Then, these SMF-28 optical fiber tapers were irradiated using 193 nm excimer laser radiation for improving their surface hydrophilicity. This irradiation process took place with the supporting SMF-28 tapered optical fiber being immersed into deionized water, through a tungsten amplitude mask for defining hydrophilic areas of typical lengths of ~ 100 μm; the total energy dose dissipated was 13 J cm^−2^. With the help of a micro-syringe silk fibroin droplets were dispersed onto the hydrophilic optical fiber tapers, on a vertical placement mode, for forming elongated cylindrical shape cavities of typical length of ~ 100 μm and diameters of ~ 33 μm; namely, a thin silk fibroin overlayer of ~ 1.5 μm thickness was casted on the glass surface. These silk fibroin cylindrical cavities were left to fully dry in ambient atmosphere, so the soft material reaching the Silk I amorphous state. Accordingly, for transforming the cavities to the crystalline Silk II structure an annealing procedure took place at 180 °C for 10 min in ambient air, resulting a nominal diameter shrinkage with shrinkage ratio 0.34%. The total tile of the annealing process for reaching the temperature of 180 °C lasted for ~ 20 min, with a typical ramping rate of 8 °C min^−1^.

### Experimental setup and spectral measurements

The experimental setup for spectrally characterizing whispering gallery mode resonation inside silk fibroin cavities the investigation of the photo-elastic behavior, is depicted in the Supplementary Part, Fig. S1. The fibroin cavity supporting mount is strained from a mechanical actuator with the ability to make steps in the micrometric scale. The ~ 30 μm diameter tapered optical fiber allows the controllable application of minimal strains along the longitudinal axis of the supporting microfiber onto the cavity; thus, warranting linear material response. For the excitation of the WGMs, a 2.2 μm diameter tapered optical fiber was used. A broadband lamp and a CMOS sensor-based camera with a 20 × magnification objective lens were used for the imaging.

The tapered optical fiber-silk fibroin cavity components were mounted on a bronze fork using UV glue (see Fig. S1), with the ability to controllably apply strain. A DC servo motor actuator was used for the precise elongation of the optical fiber with the silk fibroin cylinder, with a step of 0.2 μm. For the signal recording an optical spectrum analyzer (ANDO AQ6317B) was used, at a wavelength resolution of 10 pm, and a polarizing optical fiber (Zing™ Fibercore Ltd, UK) was used for the separation of the TE modes and TM modes that are supported in the silk fibroin resonator. The strain exerted onto the 30 μm diameter taper section was calibrated using again WGM resonation for TE and TM polarization, while assuming the Pockel’s coefficients holding for silica glass at the wavelength of propagation.

### Brillouin light scattering measurements

We used Brillouin light scattering (BLS) to determine the mechanical properties of Silk I and II by measuring the velocity of longitudinal (${c}_{L}$) and transverse (${c}_{T}$) gigahertz (GHz) acoustic waves. The experimental apparatus, shown in Fig. S2a, can be used to measure BLS in 90A or backscattering geometry. In 90A geometry the probed GHz waves have a wavevector $q=(4\pi /\lambda )\mathrm{sin}{45}^{o}=16.7\upmu {\mathrm{m}}^{-1}$, where $\lambda =532 nm$ is the laser wavelength. In backscattering BLS the probed wavevector $q=4\pi n/\lambda$, where $n$ is the refractive index of the material. All the measured BLS spectra are better represented with Gaussian peak profiles. The samples for BLS in 90° transmission (90A) geometry consisted of smooth, homogeneous films with a thickness of ca. 13.5 μm on a 1 mm-thick soda lime glass slide.

In 90A with VV polarization (Fig. S2a) the frequency of longitudinal acoustic waves is ${f}_{L,1}=8.19\mathrm{ GHz}$ and ${f}_{L,2}=8.2\mathrm{ GHz}$, and the velocities ${c}_{L,1}=(3083 \pm 10) m/s$ and ${c}_{L,2}=(3086 \pm 10) m/s$. In VH polarization (Fig. S2b) we probe transverse acoustic waves with frequency ${f}_{T,1}=4.15\mathrm{ GHz}$ and ${f}_{T,2}=4.16\mathrm{ GHz}$. The transverse velocities are ${c}_{T,1}=\left(1561\pm 10\right) m/s$ and ${c}_{T,2}=\left(1563\pm 10\right) m/s$ for Silk 1 and 2, respectively. The densities of Silk I and II are $1400 kg/{m}^{3}$ and $1450 kg/{m}^{3}$. The shear moduli ($G=p {c}_{T}^{2}$) are ${G}_{1}=\left(3.41\pm 0.05\right) GPa$ and ${G}_{2}=\left(3.54\pm 0.05\right) GPa$. The Poisson ratio $v=\left(1-0.5{c}_{L}^{2}/{c}_{T}^{2}\right)/\left(1-{c}_{L}^{2}/{c}_{T}^{2}\right)=0.328$ for both samples. The Young’s moduli are found from:

$$E = \frac{{\left( {1 + v} \right)\left( {1 - 2v} \right)}}{1 - v}pc_{L}^{2} { },$$

and they are equal with ${E}_{1}=\left(9.05 \pm 0.05\right) GPa$ and ${E}_{2}=\left(9.40 \pm 0.05\right) GPa$. The thick samples were also studied with backscattering BLS, from which we can extract the refractive index with the relationship $n=\sqrt{2}{f}^{BS}/2{f}^{90}$. At 532 nm wavelength the measured values are ${n}_{1}=1.593$ and ${n}_{2}=1.596$. In addition to the thick samples for 90A measurements, we have prepared thin films of Silk I and II using the processes described above onto CaF_2_ substrates, which resemble more the samples used in the WGM experiment. The thinner silk films could be measured with BLS in backscattering geometry (Fig. S2d) from which we extract the Young moduli of Silk I and II equal with ${E}_{1}=\left(9.08\pm 0.05\right) GPa$ and ${E}_{2}=\left(9.90\pm 0.05\right) GPa$, respectively.

In addition to the mechanical properties, BLS can be used to derive the relative values of the photo-elastic constants in Silk I and II. Although the mechanical properties are identical, the optical anisotropy per optical axis of Silk I is significantly higher than Silk II (see the intensities in Fig. S2b and c). The BLS intensity ($I$) for longitudinal waves and VV polarization is:

$$I^{VV} \propto \frac{{\left| {p_{12} } \right|^{2} }}{{c_{L}^{2} }} .$$

The BLS intensity for transverse waves and VH polarization is:

$$I^{VH} \propto \frac{{\left| {p_{44} } \right|^{2} }}{{c_{T}^{2} }}\frac{{n^{2} - \sin^{2} 45^{o} }}{{n^{2} }} ,$$

where $n$ is the refractive index and the ${p}_{11}$ and ${p}_{12}$ are photoelastic constants. Additionally, for isotropic solids we have ${p}_{44}=\left({p}_{11}-{p}_{12}\right)/2$. For the depolarization ratio we then have:

$$\frac{{I^{VH} }}{{I^{VV} }} = \left( {\frac{{f_{L} }}{{f_{T} }}} \right)^{2} \frac{{\left| {p_{44} } \right|^{2} }}{{\left| {p_{12} } \right|^{2} }}\frac{{n^{2} - \sin^{2} 45^{o} }}{{n^{2} }} ,$$

where ${f}_{L}$ and ${f}_{T}$ are the observed frequencies of the LA and TA phonons. From the relationship for ${I}^{VV}$ we get ${p}_{12}^{silk 1}/{p}_{12}^{silk 2}=1.32$. From ${I}^{VH}$ we get ${p}_{44}^{silk 1}/{p}_{44}^{silk 2}=1.23$. Finally, from ${I}^{VH}/{I}^{VV}$ and all the above we get ${p}_{11}^{silk 1}/{p}_{11}^{silk 2}=1.31$.

## Supplementary Information

Supplementary Information.

## Data Availability

The authors state that all relevant data supporting this study's findings are available upon request from the corresponding authors.
