# Supplementary material for: Photo-elasticity of silk fibroin harnessing whispering gallery modes
Source: Sci Rep. 2023 Jun 16;13:9750. doi: 10.1038/s41598-023-36400-0 (PMC10275884; doi:10.1038/s41598-023-36400-0)
Supplement: Supplementary file 1 — Supplementary Information. [file 41598_2023_36400_MOESM1_ESM.docx]

Supplementary information-

**Photo-elasticity of silk fibroin harnessing whispering gallery modes**

***Nikolaos Korakas,^1,2^ Davide Vurro,^3^  Odysseas Tsilipakos,^1,4^ Thomas Vasileiadis^5^,Bartlomiej Graczykowski^5^, Annamaria Cucinotta,^6^ Stefano Selleri,^6^ George Fytas^1,7^, Salvatore Iannotta,^3^ Stavros Pissadakis^1, *^***

*^1^ Foundation for Research and Technology-Hellas (FORTH), Institute of Electronic Structure and Laser (IESL), 70013 Heraklion, Greece*

*^2^Department of Materials Science & Technology, University of Crete, 70013, Heraklion, Greece.*

*^3^ Institute of Materials for Electronics and Magnetism (IMEM), CNR, 43124 Parma, Italy*

*^4^* *Theoretical and Physical Chemistry Institute, National Hellenic Research Foundation, GR-11635 Athens, Greece*

*^5^ Adam Mickiewicz University, 61-712 Poznań, Poland*

*^6^ University of Parma, 43121 Parma, Italy*

*^7^ Max Planck Institute for Polymer Research, Ackermannweg 10, 55128 Mainz, Germany*

*^*^Corresponding author:* [pissas@iesl.forth.gr](mailto:author_three@uni-jena.de)

1. Experimental setup


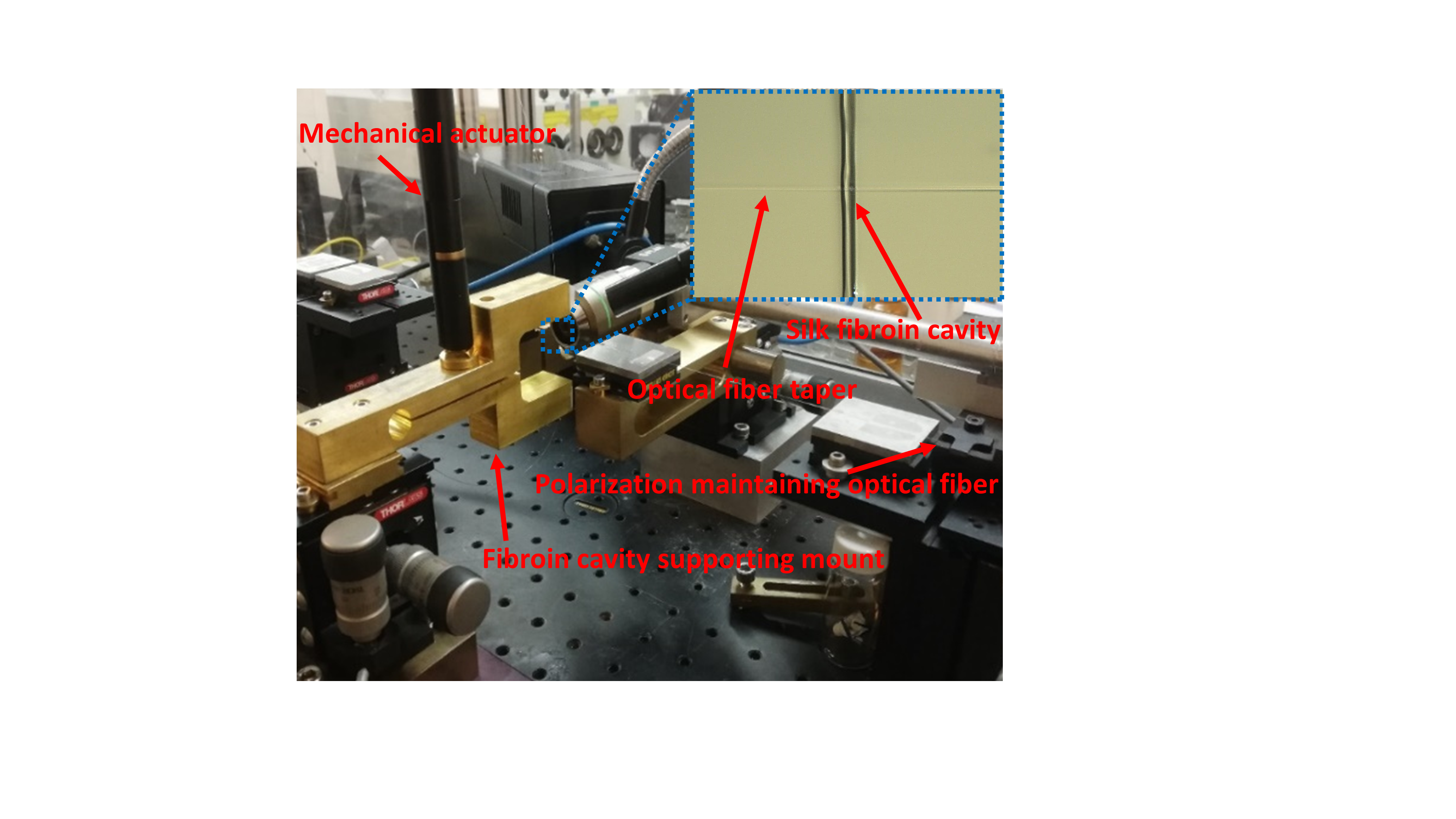


**Figure S1.** Whispering gallery modes experimental setup for the photo-elastic measurements of silk fibroin cavities. Inset Image from CMOS based camera that depicts the silk fibroin cavity and the optical fiber taper injecting light (through the evanescent field) inside the cylindrical resonator.

1. Brillouin Light Scattering measurements


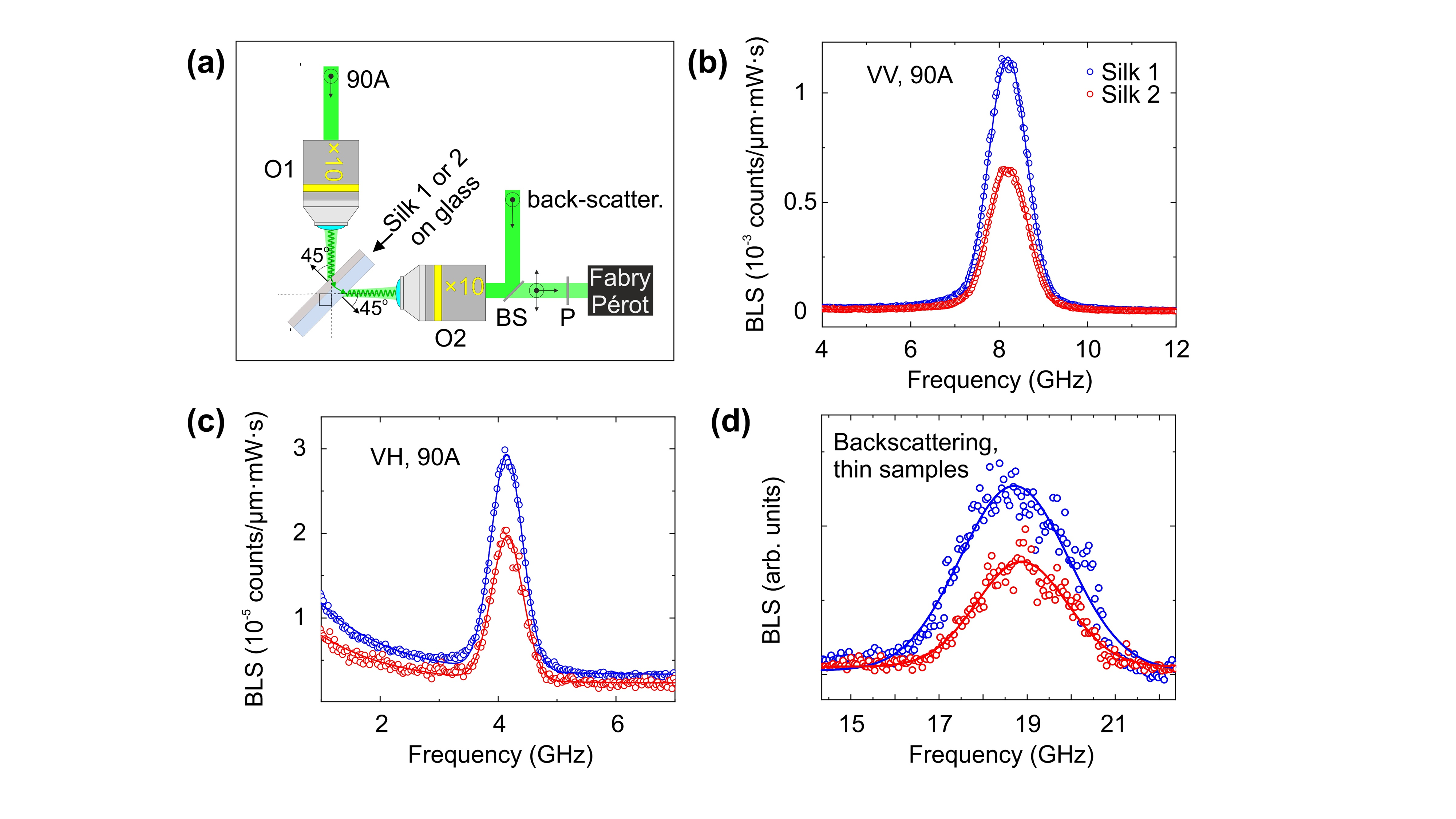


**Figure S2.** (a)The experimental setup used to measure Brillouin light scattering spectra for Silk 1 (blue) and 2 (red) in (b) 90o transmission geometry with VV polarization, (c) 90o transmission with VH polarization, and (d) thin samples resembling the WGM experiment in backscattering geometry with VV polarization. The spectra are represented with Gaussian peak profiles (solid lines). The spectra in (b) and (d) are normalized with the incident power, the exposure time, and the film thicknesses (13.5 and 13.3 μm for Silk 1 and 2, respectively).

1. Details of simulations for mode allocation in silk fibroin cavities

The calculation of the whispering gallery modes in the system under study was conducted with the use of full wave eigenvalue simulations utilizing COMSOL Multiphysics. In the simulations, the silk fibroin cavity was considered perfectly cylindrical, and treated as a two-dimensional resonant structure. This is justified by the slowly-varying geometry of the cavity along the *z* axis, and the fact that modal excitations take place within its middle section, being far from side-located turning points. The resonant modes are described by two indices (the azimuthal (*m*) and radial (*l*) orders) and their polarization (TE: **H**=*H*_z_**z** and TM: **E**=*E_z_***z** with reference to Figure 1 of the manuscript).

- 1. **Verification of numerical tool**

Before employing the numerical simulations in the silk fibroin cavities under study, their accuracy was verified in an archetypical cylinder resonator geometry by comparing the simulation results against rigorous analytical solutions^1^. A homogeneous cylinder with *n*_cyl_=1.513, *n*_host_=1 and *r*=15.8 μm is considered and the comparison in calculated resonant wavelength between theory and simulation for an indicative TE (*m*=89) and TM (*m*=89) resonant mode is presented in Table S1. Excellent agreement is observed with the fractional error in resonant frequency being below 1%.

The transcendental equations that are solved are^1^ :

for the TE (**H**=*H*_z_**z**) and

for the TM (**E**=*E_z_***z**) polarization, respectively, where $u=k_{\text{cyl}}r$ and $v=k_{\text{host}}r$, with $k_{\text{cyl}}=k_{0}n_{\text{cyl}}$ and $k_{\text{host}}=k_{0}n_{\text{host}}$. For each azimuthal order *m*, each equation admits a denumerable infinite number of roots corresponding to modes of different radial order *l.*

**Table S1**. Comparison of calculated resonant frequencies between analytical and simulation results for the archetypical dielectric cylinder structure depicted in the inset^2^.

| **Mode** | **λ_0_ – theory  (nm)** | **λ_0_ – simulation  (nm)** | **Fractional error** | 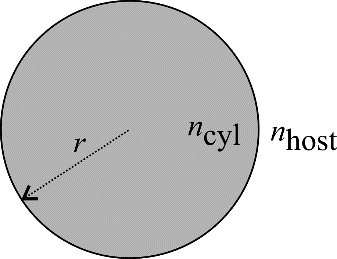 |
| --- | --- | --- | --- | --- |
| TE, *m*=89 | 1562.8 | 1562.7 | -0.6% |
| TM, *m*=89 | 1551.0 | 1550.9 | -0.6% |

- 1. **Simulation results for the silk fibroin cavity**

The system under study is depicted in Figure S3(a). It is a two-dimensional equivalent of the actual structure. The parameters are $n_{\text{Silk} \text{I}}=1.5215$, $n_{\text{SiO}_{2}}=1.45$, $r_{\text{out}}=15.78 \text{µm}$, $r_{\text{inn}}=14.2 \text{µm}$. Modes of different azimuthal and radial order can be supported by the structure, identified by the azimuthal order (*m*) radial order (*l*). Indicative examples of first-radial order modes of the TE polarization are shown in Figure S3(c-d). The free spectral range is ~16 nm in close agreement with the measurement. Based on the calculated resonant wavelengths, we determine the mode allocation in Figure 2 of the manuscript. Examples of higher-radial-order resonances are depicted in Figure S3(e-f). The resonant wavelengths can be associated with secondary spectral features of the experimental transmission curve^3^, as seen in Figure S3(b). Note that higher-radial-order modes extend further into the cavity; they thus experience a shorter effective circumference and a lower group index environment. As a result, they exhibit larger FSR values compared to modes of first radial order $\Delta\lambda_{FSR}\propto(n_{g}L_{\text{eff}})^{-1}$ .


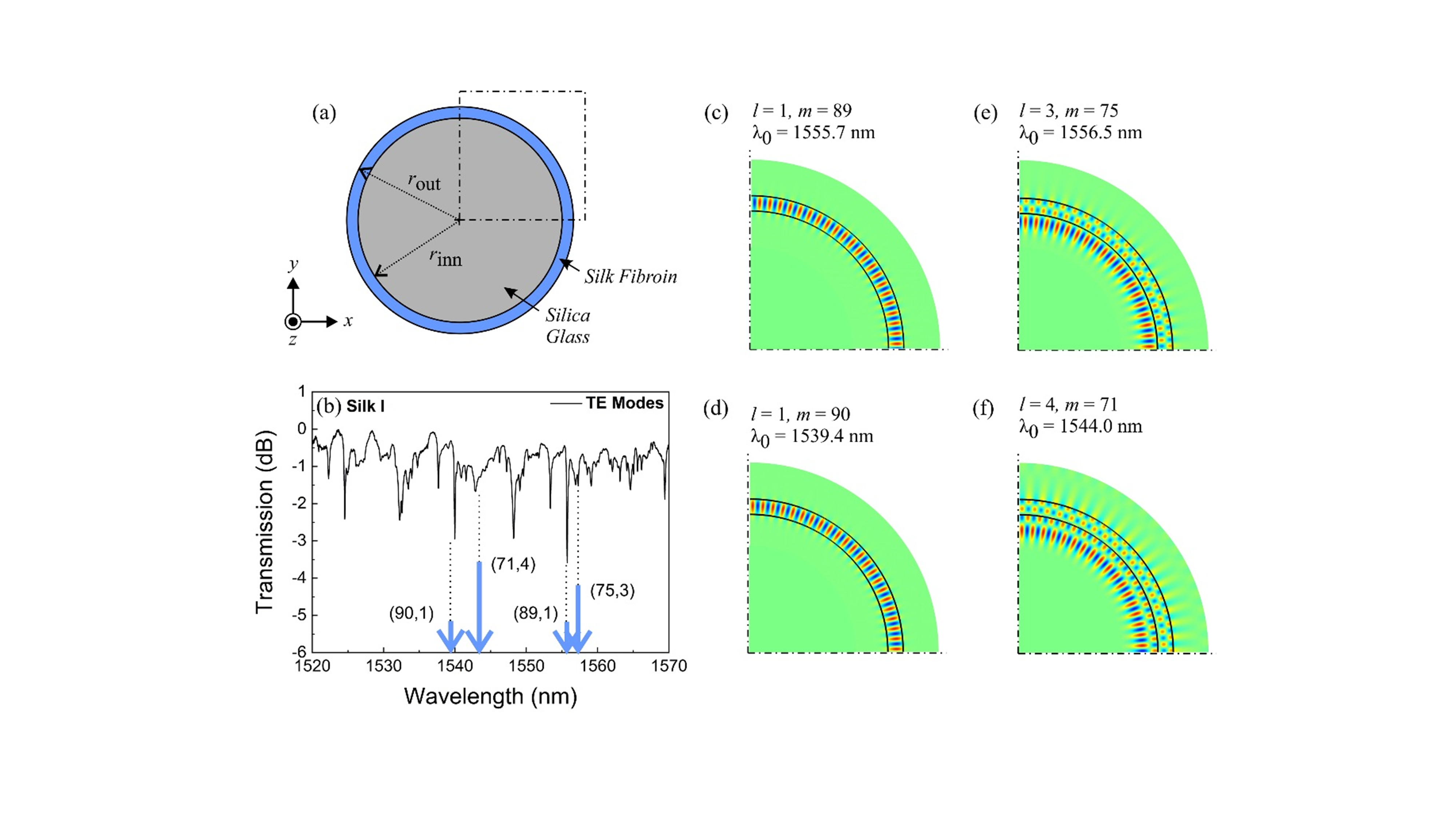
 **Figure S3**. (a) Schematic of resonant silk fibroin cavity under study. A cylindrical structure is assumed, invariant along z, modeled as a 2D geometry. (b) Experimentally measured transmission spectra for TE polarization, reproduced from Figure 2 in the manuscript. (c-f) Mode profiles for indicative resonances of first(c,d), third (e), and fourth (f) radial order (l). The simulated resonant wavelength is marked in panel (b) and found in very good agreement with the spectral locations of the experimental features.

- 1. **Filed confinement calculations**

Light confinement in the silk layer was assessed by integrating the stored energy density inside the different material regions, as a post-processing step in the finite element method eigenvalue simulations. This is an important calculation so as to determine if the elasto-optic parameters of the silica glass affect the experimental measurements. For the TE mode of Silk I with azimuthal order *m*=89 which is depicted in Figure S2(c), only 2.1% of the total stored energy resides in the silica glass, 97.1% in the silk layer, and the remaining 0.8% in the surrounding air. The corresponding percentage in silica for the first-radial order TM mode is very similar: approximately 1.2%. As a result, for the first-radial-order modes which are excited in the structure (see Figure 2 in the manuscript), almost all the injected light intensity will be waveguided through the silk fibroin region. Therefore, the contribution of the silica glass in the photo-elastic behavior is negligible and can be ignored. For higher radial orders, the modes become more leaky and gradually light resides further into the silica region [see Figure S2(e-f)]. Specifically, for modes of radial order *l* = (2,3,4) the stored energy in silica is in the order of (32%, 67%, 71%), respectively, and in air (1.8%, 2.5%, 6.1%).

1. Spectral measurements of Silk II WHM cavities under longitudinal strain application

Spectral measurements for the WGM Silk II cavity under longitudinal strain application are presented in Figure S4; this characterization procedure followed the initial measurements of the WGM metastable Silk I cavity.


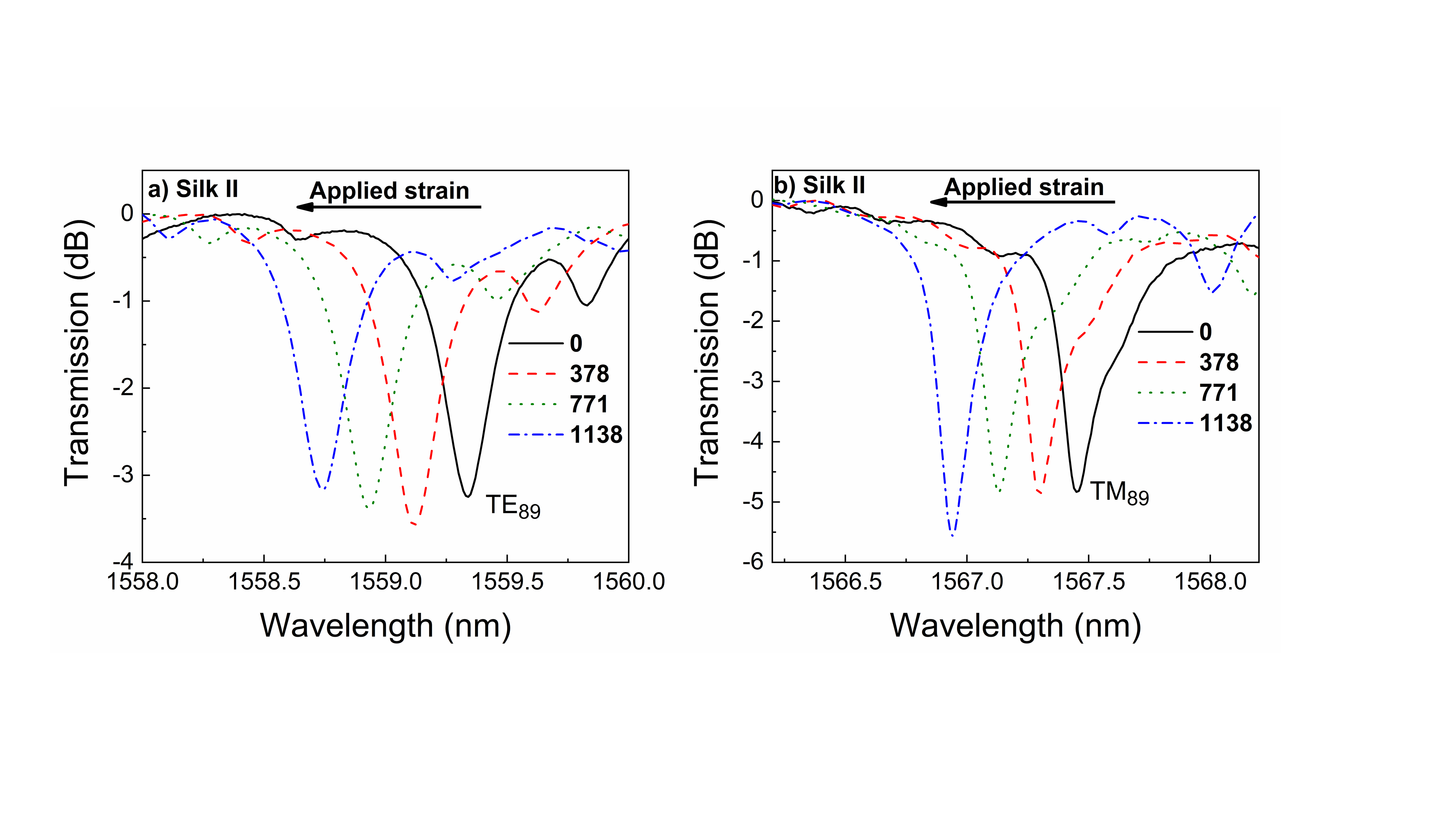


**Figure S4.** Transmission spectra of the TE and TM WGMs with modal order m=89, confined inside the silk fibroin cavity for Silk II state, under applied strain along the longitudinal axis of the supporting optical fiber taper

1. Finite element analysis of the component’s strain tensors

A mechanical strain simulation was carried out with the assistance of a finite element analysis program that is available commercially. The strain components along the z (the longitudinal axis) and t (transversal x- and y-axes) directions were conducted to determine Pockel’s coefficients. In addition, it was essential to specify the component’s actual Poisson ratio. We have already calculated the Poisson ratio based on BLS measurements for the silk fibroin material in the form of a thick layer. We use this value (v=0.328) as a simulation input. The ε_z_ strain values used in these calculations were directly deduced from the experimental data available. Specifically, the ε_z_ component (Figure S5(a)) represents the strain applied in the optical fiber’s long axis. The e_z_= 3922 με strain value was used as a stimulus input to the simulation. Because of computational limitations, a scaling down at the length of the supporting optical fiber beam was made, reducing its total length from 37.5 mm to 400 μm. This reduction was made without altering the length of the silk fibroin cavity, which was maintained at 100 μm. The rest of the geometric dimensions are the same as the previous sections for the silk cavity and the supporting glass beam. The distribution strain components for Silk I are presented in Figure S5. Τhe ε_t_ component (Figure S5(b)) is the induced strain on the transverse (vertical) axis with e_t_=1118 με strain value resulting from the simulation. The value can be considered absolute because it was measured at multiple points within the cylindrical cavity of Silk. When applying the formula for Poisson’s ratio, v= et/ez, we get the Silk component Poisson ratio value v=0.285.


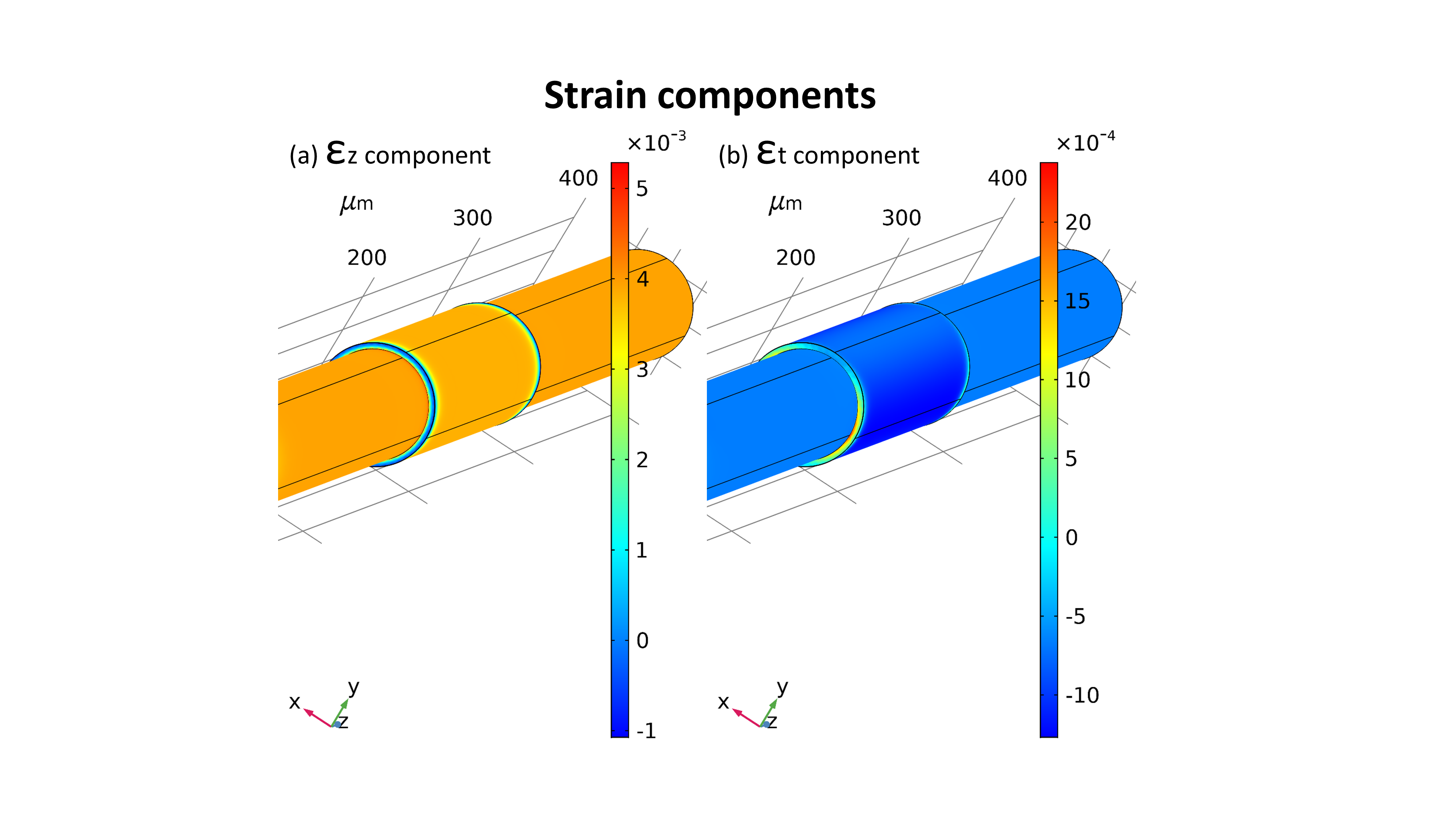


**Figure S5.** Finite element strain tensor analysis of the Silk I cylindrical shape resonator. The εz component (a) represents the strain that is applied in the long axis of the optical fiber with 3922 με strain value. On the contrary, εt component (b) is the induced strain on the transverse (vertical) axis with 1118 με strain value.

1. Raman measurements

To investigate the crystallization process from the Silk I to the Silk II structure Raman measurements of Silk I and II were performed using a 532nm frequency double Nd:YAG, operating at 24mW, while focused on samples using an inverse microscope system and a 50X times objective lens. Exposure time was 5sec, while 5 measurements were taken per spot^4^. Raman spectra for a WGM cavity in Silk I and II structure are presented in Figure S5.





**Figure S6.** Raman spectra of Silk I and II WGM cavities, obtained using 532nm laser excitation.

The peak of interest with respect to the crystallization of β-sheets is the 1262cm^-1^, 1236 cm^-1^ (Amide III) and at 1085cm^-1^, which appears apparently amplified -by a factor of X2- in the Silk II measurement with respect to the Silk I. The peak at 1660cm^-1^ is attributed to the Amide I structure^5^.

**References**

1. Stratton , J. A. *Electromagnetic theory*. (McGraw-Hill Book Company, Inc., 1941).

2. Tsilipakos, O., Yioultsis, T. V & Kriezis, E. E. Theoretical analysis of thermally tunable microring resonator filters made of dielectric-loaded plasmonic waveguides. *J. Appl. Phys.* **106**, 93109 (2009).

3. Tsilipakos, O. & Kriezis, E. Microdisk resonator filters made of dielectric-loaded plasmonic waveguides. *Opt. Commun.* **283**, 3095–3098 (2010).

4. Katsara, K., Kenanakis, G., Viskadourakis, Z. & Papadakis, V. M. Polyethylene Migration from Food Packaging on Cheese Detected by Raman and Infrared (ATR/FT-IR) Spectroscopy. *Mater. (Basel, Switzerland)* **14**, (2021).

5. Monti, P., Freddi, G., Bertoluzza, A., Kasai, N. & Tsukada, M. Raman spectroscopic studies of silk fibroin from Bombyx mori. *J. Raman Spectrosc.* **29**, 297–304 (1998).
